# An Observational Study of Indian Medical Students: Are We Truly Aware of Monkeypox?

**DOI:** 10.7759/cureus.43952

**Published:** 2023-08-22

**Authors:** Sarita K Sharma, Chinmay Guralwar, Rashi Mahajan, Ujwala U Ukey

**Affiliations:** 1 Department of Community Medicine, Government Medical College and Hospital, Nagpur, Nagpur, IND

**Keywords:** prevention, transmission, monkeypox, vaccination, awareness

## Abstract

Introduction

Monkeypox is a zoonotic disease caused by an enveloped double-stranded DNA virus from the Poxviridae family. As the future front-liners of healthcare, it is crucial to equip medical students with adequate knowledge of diseases like monkeypox that pose a potential pandemic threat.

Aim

This study was planned to evaluate the level of awareness of monkeypox among Indian medical students.

Methodology

This was a cross-sectional, questionnaire-based research conducted using a web-based platform. The study population consisted of medical (MBBS) students from India studying in their first, second, third, final, and internship years. Data collection was done using a self-designed, semi-structured questionnaire.

Results

Out of the 511 students who filled out the form, 280 (54.79%) were males and 230 (45.01%) were females. Of the study respondents, 459 (89.82%) had heard about monkeypox. The internet (60.5%) and social media (55.3%) were the most common sources of information about monkeypox. The study participants were found to be fairly aware of the different modes of monkeypox transmission. A significant majority (about three-fourths) of internship-year students demonstrated knowledge about preventive measures for monkeypox, with only a minimal 5% reporting a lack of awareness. In contrast, a substantial percentage of first-year students (38%) and second-year students (37.6%) admitted to being unaware of prevention methods, with a statistically significant difference (p<0.001).

Conclusion

The overall knowledge levels were generally satisfactory, with respondents demonstrating awareness of different modes of monkeypox transmission. However, a concerning proportion of first-year (38%) and second-year (37.6%) students reported being unaware of prevention methods.

## Introduction

Monkeypox is a zoonotic disease caused by an enveloped double-stranded DNA virus from the Poxviridae family. The disease was first identified in humans in 1970 in the Democratic Republic of the Congo. Since then, human cases have been reported in 11 African countries. In May 2022, multiple cases of monkeypox were identified in several non-endemic countries [1]. The disease primarily occurs in forested rural areas of Central and Western Africa. Although there is a higher risk of this disease in endemic areas, it remains under-recognized and underreported [2]. The first case of monkeypox in the WHO South-East Asia Region was reported in India, in a 35-year-old man who had arrived from the Middle East [3]. Disease transmission can occur from infected animals through direct contact with blood, body fluids, or cutaneous or mucosal lesions. It can also spread from human to human due to close contact with respiratory secretions, skin lesions of an infected person, or objects and fomites recently contaminated. Monkeypox presents a clinical picture similar to smallpox. It can lead to complications such as secondary infections, bronchopneumonia, sepsis, encephalitis, and corneal infections, potentially resulting in vision loss [1]. One of the major challenges in preventing monkeypox disease is a lack of knowledge, particularly among healthcare workers [4]. A study conducted in Indonesia to assess the knowledge of monkeypox among general practitioners (GPs) found an overall low level of knowledge among the GPs [5]. Being the future of the healthcare front, it is crucial to equip medical students with adequate knowledge of diseases like monkeypox, which pose a pandemic-level threat. Increasing knowledge of this disease is vital for enhancing the capacity of GPs to respond to human monkeypox cases and to report them to a disease surveillance system. Frontline healthcare workers need to be aware of this and should consistently take travel histories. Individuals presenting with a fever and rash should be asked additional questions [4]. This study was designed to evaluate the awareness of monkeypox among Indian medical students.

## Materials and methods

Study setting

This study was conducted from August to September 2022 as a cross-sectional, questionnaire-based research using a web-based platform.

Study population

The study population consisted of medical (MBBS) students from various regions of India studying in their first, second, third, final, and internship years.

Inclusion and exclusion criteria

The inclusion criteria for participants were as follows: being enrolled in any academic year of MBBS or undergoing a Compulsory Rotatory Residential Internship (CRRI) and being aged 18 years or above. The exclusion criterion was a refusal to provide informed consent. Participants who met the selection criteria were given a detailed briefing about the study, accompanied by an informed consent form attached to the questionnaire.

Sample size

The sample size was estimated based on the assumption that the rate of awareness about monkeypox among Indian medical students is 50%. Considering a non-response rate of 10%, a minimum required sample size of 425 participants was estimated. However, 516 participants filled out the Google Form, of which five participants did not consent for their data to be used. Finally, data from 511 participants was analyzed. For most of these students, no class specifically on monkeypox was held.

Ethical aspects

Ethical approval from the Institutional Ethics Committee was obtained before initiating the study as per letter number EC/Pharmac/GMC/NGP/3833. Participants were ensured of complete confidentiality and anonymity.

Data collection tool

Data collection was done using a self-designed, semi-structured questionnaire with three parts. Part I collected the demographic characteristics of the participants, such as age and year of study. Part II assessed knowledge about the causative organism and the disease, while Part III explored awareness of treatment and prevention of the disease. The questionnaire was formulated after reviewing several articles on monkeypox [6,7].

The questionnaire was evaluated for content validity by community health experts, wherein the data collection instrument was tested to determine if it covered all relevant aspects of monkeypox. However, no specific validity test was used. The questionnaire, thus devised, was pre-tested by collecting data from 10 students to check for comprehensiveness and clarity. An entire list of students was procured, and from that, two students were randomly selected from each year of MBBS and interns. Based on the results of the pilot study, appropriate modifications were made to finalize the questionnaire.

Statistical analysis

Data are summarized as means and SDs for continuous variables and as frequencies and percentages for categorical variables. Evaluation of the association between categorical variables is done using the Chi-square test.

P-values less than 0.05 are considered to be statistically significant. All hypotheses are formulated using two-tailed alternatives against each null hypothesis. Statistical data analyses are done using JASP, version 0.17.1.0.

## Results

Table 1 shows the socio-demographic characteristics of the study population. Out of 511 students who responded, 280 (54.79%) were males. The mean age of the participants was 21.17±1.48 years. Most participating students were from government medical colleges (91.39%) and residing in urban areas (82.39%). These government medical colleges were spread across the country, thereby ensuring nationwide coverage. There was also some representation from private and semi-government colleges. About half (54.45%) of the students were living in the hostel, while 36.7% lived at home. The study participants were from the states of Maharashtra, Uttar Pradesh, Odisha, Rajasthan, Delhi, Madhya Pradesh, Chattisgarh, West Bengal, and Haryana.

**Table 1:** Socio-demographic characteristics of students who participated in the study (n=511).

Variable	Category	Number	Percentage
Gender	Male	280	54.79
	Female	230	45.01
	Prefer not to say	1	0.2
Year of work/study	First	122	23.87
	Second	127	24.85
	Third	81	15.85
	Final	81	15.85
	Internship	100	19.57
Type of institute of study	Government	467	91.39
	Private	44	8.61
Residence	Urban	421	82.39
	Rural	90	17.61
Living in	Hostel	278	54.4
	Home	188	36.79
	Other	45	8.81
Have you ever heard of Monkeypox?	Yes	459	89.82
	No	52	10.18

**Figure 1:**
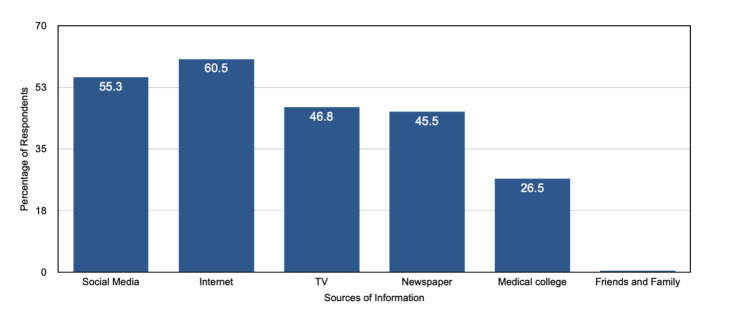
Sources of information about monkeypox.

Out of the total study respondents, 459 (89.82%) had heard about monkeypox, of which 92 (75.2%) and 109 (86.5%) participants were from the first and second years, respectively, while the participants from third, final, and internship years were 78 (96.3%), 81 (100%), and 98 (98%), respectively. This difference was statistically significant (p<0.001). Figure 1 shows different sources from which the participants learned about monkeypox.

Internet (60.5%) and social media platforms such as WhatsApp, Facebook, and Instagram (55.3%) were the most common sources, followed by television (46.8%), newspapers (45.5%), and medical college (26.5%). Friends and family (0.4%) were the least common sources of information.

As seen in Table 2, questions number 1 to 8 tested the students' knowledge about monkeypox, while questions 9 to 12 focused on monkeypox prevention and treatment.

**Table 2:** Frequency of correct responses stratified by the year of study - knowledge questions.

Sr No.	Knowledge Questions	Correct response	Year of study					Total correct response (%)	χ² (df)	P- value
			First (n=92)	Second (n=109)	Third (n=78)	Final (n=81)	Internship (n=98)			
			Correct answer frequency (%)							
1	The Monkeypox virus belongs to which family of viruses?	Poxviridae	46 (50.0)	58 (53.2)	37 (47.4)	25 (30.9)	43 (43.9)	209 (45.5)	38.39 (20)	0.007
2	When was the first human case of Monkeypox disease recorded?	1970	29 (31.5)	47 (43.1)	43 (55.1)	53 (65.4)	65 (66.3)	237 (51.6)	63.09 (12)	<0.001
3	Where was the first human case of Monkeypox disease recorded?	Democratic Republic of the Congo	50 (54.3)	75 (68.8)	62 (79.5)	62 (76.5)	71 (72.4)	320 (69.7)	28.16 (12)	0.005
4	What are the hosts of Monkeypox virus?	Rodents,Non human primates and humans	42 (45.7)	48 (44.0)	39 (50.0)	32 (39.5)	45 (45.9)	206 (44.8)	26.0 (16)	0.050
5	What is the period of maximum infectivity for Monkey pox disease?	From development of symptoms until the rash has fully healed and a fresh layer of skin has formed	27 (29.3)	29 (26.6)	25 (32.1)	38 (46.9)	40 (40.8)	159 (34.6)	70.02 (16)	0.009
6	Immuno-compromised individuals are more susceptible to monkey pox	True	72 (78.3)	84 (77.1)	43 (55.1)	34 (42.0)	60 (61.2)	293 (63.8)	66.1 (2.8)	<0.001
7	What is the incubation period of Monkey pox?	7-14 days	37 (40.2)	50 (45.9)	38 (48.7)	36 (44.4)	56 (57.1)	217 (47.2)	71.96 (12)	<0.001
8	By what method will you confirm the diagnosis of Monkey pox?	PCR	73 (79.3)	87 (79.8)	45 (57.7)	31 (38.2)	53 (54.0)	289 (62.9)	49.3 (6.4)	<0.001
		Culture	25 (27.1)	45 (41.3)	35 (44.8)	50 (61.7)	45 (45.9)	200 (43.5)	21.4 (1.4)	<0.001
	Disease prevention and treatment questions									
9	What is the preferred vaccine to protect against monkeypox?	JYNNEOS	27 (29.3)	27 (24.8)	41 (52.6)	32 (39.5)	37 (37.8)	162 (35.2)	94.74 (12)	<0.001
10	What is the route of administration of the preferred vaccine?	Subcutaneous	26 (28.3)	37 (33.9)	23 (29.5)	19 (23.5)	24 (24.5)	129 (28.1)	26.21 (12)	0.01
11	Which age group should get the Monkeypox vaccine?	18 years and above	24 (26.1)	35 (32.1)	42 (53.8)	50 (61.7)	52 (53.1)	204 (44.4)	99.85 (16)	<0.001
12	What is the available pharmacological treatment of Monkey pox?	Tecovirimat (TPOXX)	36 (39.1)	32 (29.3)	32 (41.0)	37 (45.6)	56 (57.1)	193 (42.0)	17.15 (4)	0.001
		Cidofovir	10 (10.8)	12 (11.0)	33 (42.3)	42 (51.8)	35 (35.7)	132 (28.7)	61.44 (4)	<0.001
		Brincidofovir	12 (13.0)	17 (15.5)	28 (35.8)	42 (51.8)	36 (36.7)	135 (29.4)	45.59 (4)	<0.001

The frequency of correct responses about the mode of transmission of monkeypox is shown in Table 3.

**Table 3:** Knowledge items of modes of transmission of monkeypox.

Modes of transmission	Correct response	Year of study					Total correct response (%)	χ² (df)	P-value
		First (n=92)	Second (n=109)	Third (n=78)	Final (n=81)	Internship (n=98)			
		Correct answer frequency (%)							
Touching objects, fabrics (clothing, bedding, or towels), and surfaces that have been used by someone with monkeypox.	Yes	40 (43.5)	59 (54.1)	48 (61.5)	65 (80.2)	71 (72.4)	283 (61.6)	38.07 (8)	<0.001
Direct contact with monkeypox rash, scabs, or body fluids from a person with monkeypox	Yes	66 (71.7)	82 (75.2)	40 (51.3)	27 (33.3)	49 (50.0)	264 (57.5)	58.37 (8)	<0.01
Contact with respiratory secretions.	Yes	56 (60.9)	67 (61.5)	15 (19.2)	45 (55.6)	64 (65.3)	275 (59.9)	22.44 (8)	0.004
Direct contact during sexual activities.	Yes	47 (51.1)	50 (45.9)	21 (26.9)	28 (34.6)	40 (40.8)	186 (40.5)	22.39 (8)	0.004
Mother to foetus transmission via placenta(vertical transmission).	Yes	40 (43.5)	55 (50.5)	30 (38.5)	32 (39.5)	35 (35.7)	192 (41.8)	21.84 (8)	0.005
Zoonotic Transmission.	Yes	39 (42.4)	58 (53.2)	37 (47.4)	23 (28.4)	43 (43.9)	200 (43.5)	40.31 (8)	<0.001
Droplet nuclei	Yes	34 (37.0)	54 (49.5)	37 (47.4)	37 (45.7)	53 (54.1)	215 (46.8)	14.45 (8)	0.07
Mosquito bite	No	29 (31.5)	49 (45.0)	40 (51.3)	29 (35.8)	41 (41.8)	202 (44.0)	16.34 (8)	0.04

The data in Table 4 illustrates the response to various symptoms of monkeypox stratified by the year of study. Only 4.1% and 1.2% of internship and final-year students, respectively, answered that they did not know about the symptoms of monkeypox. In contrast, 21.7% of first-year and 18.3% of second-year students said that they did not know about it, and the difference was statistically significant (p=<0.001).

**Table 4:** Symptoms of monkeypox.

Symptoms	Correct response	Year of study					Total correct response (%)	χ² (df)	P-value
		First (n=92)	Second (n=109)			Internship (n=98)			
		Correct answer frequency (%)							
Fever	Yes	62 (67.4)	79 (72.5)	38 (48.7)	23 (28.4)	55 (56.1)	257 (55.9)	43.61 (4)	<0.001
Headache	Yes	57 (62.0)	70 (64.2)	40 (51.3)	38 (46.9)	64 (65.3)	269 (58.6)	9.95 (4)	0.04
Muscle pain and back ache	Yes	46 (50.0)	72 (66.1)	39 (50.0)	46 (56.8)	62 (63.3)	265 (57.7)	8.52 (4)	0.07
Swollen lymph nodes	Yes	42 (45.7)	60 (55.0)	43 (55.1)	48 (59.3)	61 (62.2)	254 (55.3)	5.89 (4)	0.21
Chills	Yes	44 (47.8)	58 (53.2)	31 (39.7)	45 (55.6)	42 (42.9)	220 (47.9)	6.21 (4)	0.18
Diarrhea	No	66 (71.7)	80 (73.4)	52 (66.7)	52 (64.2)	66 (67.3)	316 (68.8)	2.50 (4)	0.64
Exhaustion	Yes	35 (38.0)	34 (31.2)	29 (37.2)	30 (37.0)	33 (33.7)	161 (35.0)	1.45 (4)	0.84
Respiratory symptoms	No	39 (42.4)	45 (41.3)	23 (29.5)	23 (28.4)	36 (36.7)	292 (63.6)	6.41 (4)	0.17
Rash (located on genital or near it; can also be present elsewhere)	Yes	56 (60.9)	61 (56.0)	31 (39.7)	17 (21.0)	46 (46.9)	211 (45.9)	34.2 (4)	<0.001
I don’t know	0	20 (21.7)	20 (18.3)	6 (7.7)	1 (1.2)	4 (4.1)	51 (11.1)	30.07 (4)	<0.001

When asked about how to prevent monkeypox (Table 5), students in the internship year were very well aware. About three-fourths of these students answered questions 1, 2, and 3 correctly. Also, only 5% of them answered that they did not know about the ways of prevention. In contrast, 38% of first-year and 37.6% of second-year students said they did not know about it, and the difference was statistically significant (p=<0.001).

**Table 5:** Ways of prevention of monkeypox.

Ways of prevention	Correct response	Year of study					Total correct response (%)	χ² (df)	P-value
		First (n=92)	Second (n=109)			Internship (n=98)			
		Correct answer frequency (%)							
Avoid close, skin-to-skin contact with people who have a rash that looks like Monkeypox.	Yes	55 (59.8)	67 (61.5)	59 (75.6)	56 (69.1)	74 (75.5)	311 (67.7)	9.66 (4)	0.05
Do not touch the rash or scabs of a person with Monkeypox.	Yes	47 (51.1)	63 (57.8)	58 (74.4)	58 (71.6)	76 (77.6)	302 (65.7)	21.76 (4)	<0.001
Avoid contact with objects and materials that a person with Monkeypox has used.	Yes	35 (38.0)	41 (37.6)	56 (71.8)	53 (65.4)	74 (75.5)	259 56.4)	53.05 (4)	<0.001
Do not share eating utensils or cups with a person with monkeypox.	Yes	36 (39.1)	37 (33.9)	40 (51.3)	46 (56.8)	61 (62.2)	220 (47.9)	22.34 (4)	<0.001
Do not handle or touch the bedding, towels, or clothing of a person with Monkeypox.	Yes	36 (39.1)	48 (44.0)	39 (50.0)	34 (42.0)	43 (43.9)	200 (43.5)	2.14 (4)	0.71
Avoid sexual contact with infected person.	Yes	39 (42.4)	44 (40.4)	27 (34.6)	20 (24.7)	32 (32.7)	162 (35.2)	7.55 (4)	0.11
Wash your hands often with soap and water or use an alcohol-based hand sanitizer, especially before eating or touching your face and after you use the bathroom.	Yes	38 (41.3)	52 (47.7)	31 (39.7)	24 (29.6)	43 (43.9)	188 (40.9)	6.74 (4)	0.15
I don’t know	0	27 (29.3)	34 (31.2)	5 (6.4)	0 (0%)	5 (5.1)	71 (15.4)	61.8 (4)	<0.001

## Discussion

As the world continues to grapple with the consequences of the COVID-19 pandemic, it has become increasingly evident that preparedness for future health crises is imperative, particularly for those studying medicine. The first crucial step towards preparedness is awareness. Monkeypox, a rare viral disease with potential for outbreaks, poses a threat to public health. Understanding the level of awareness among medical students is essential in devising effective strategies for the early detection, diagnosis, and management of monkeypox cases. By shedding light on the awareness levels of medical students, this study aims to contribute valuable insights to the existing knowledge on preparedness for emerging infectious diseases and aid in strengthening the capacity of future healthcare providers to manage such health crises. To our knowledge, this is the first study undertaken to evaluate the level of awareness regarding monkeypox among MBBS students in India. In our study, there was almost an equal distribution of students across their year of study at the medical school, with 122 (23.87%), 127 (24.85%), 81 (15.85%), 81 (15.85%), and 100 (19.57%) students in first, second, third, final, and internship year, respectively. Out of the total participants, 459 (89.82%) participants had heard about monkeypox, whereas in the study conducted by Kaur A et al. [8], one-fourth (24.8%) of the subjects had never heard about monkeypox. It was also found that a greater percentage of participants in their third, final, and internship years had heard about monkeypox compared to first and second-year respondents. While medical colleges should have been the most common sources of information about monkeypox, it is evident from our research findings that social media and the Internet has become the commonest source. This finding was consistent with research conducted on dental students [8], where the Internet was the source of monkeypox in over 40% of respondents. The Internet truly has now become indispensable to medical education, as Le T et al. [9] had predicted. A total of 12 multiple-choice questions about knowledge of monkeypox were asked to study participants, and we found no clear trends in correct response patterns. While final year and internship students involved in more clinical aspects of medical education could answer a few questions correctly in far greater numbers than their juniors, another trend of third-year students answering a few questions correctly in greater numbers than any of their counterparts was also seen. This might have been due to the fact that third-year students have to appear for the community medicine examination and may have been taught about monkeypox in their college lectures or may have read about it on their own. This is inconsistent with the findings of Kaur A et al. [8], where higher knowledge levels were significantly related to education level. It is essential to know about the modes of transmission of any infectious disease to curb its spread. The study participants were found to be fairly aware of the different modes of monkeypox transmission. No clear trend was seen when the findings were stratified by the year of study.

There was a noticeable disparity in the level of awareness among students of different academic years regarding the symptoms and prevention of monkeypox. A significant majority (about three-fourths) of internship year students demonstrated knowledge about preventive measures for monkeypox, with only a minimal 5% reporting lack of awareness. In contrast, a substantial percentage (38%) of first-year students and (37.6%) of second-year students admitted to being unaware of prevention methods, with a statistically significant difference (p=<0.001). This discrepancy may be attributed to the greater exposure to hospital settings and clinical experience among internship-year students, contributing to their higher knowledge of monkeypox symptoms and prevention.

As evident from our research, MBBS students lacked knowledge of monkeypox in various domains, and these findings were also seen in the studies conducted on dental students [8] and physicians [10].

It is seen that behavior that is adaptive and responsive to the presence of disease can play a crucial role in mitigating the size and impact of an epidemic outbreak. However, it is not merely the existence of the disease itself but rather the awareness of its presence that triggers changes in human behavior. This highlights the importance of timely and accurate dissemination of disease information to promote informed decision-making and behavioral adjustments that can help curb the spread of diseases in populations [11]. This makes awareness of monkeypox even more important, given that WHO had declared it a Public Health Emergency of International Concern (PHEIC) [12].

Limitations

Nonetheless, it is vital to acknowledge the limitations of this study, including its cross-sectional design and reliance on self-reported data. Moreover, it was an online survey which might have compromised the validity of the responses. However, the authors have taken enough precautions to minimize these limitations by maintaining the quality of the data collection. Hence the findings of this study can be generalized to other similar settings.

## Conclusions

In conclusion, this study sheds light on the knowledge and awareness of monkeypox among medical students, revealing that most participants had heard about this new disease, with the internet and social media being the most common sources of information. The overall knowledge levels were generally satisfactory, with respondents demonstrating awareness of different modes of transmission. However, a concerning aspect is that a sizeable proportion of preclinical students reported being unaware of methods of prevention and control in comparison with the students from clinical years. This study offers valuable contributions to the literature and provides insights that can guide future efforts to enhance awareness and knowledge about monkeypox among healthcare professionals and the wider community. Continued research in this area can help formulate effective strategies to bolster the knowledge and preparedness of medical students, ultimately leading to improved public health outcomes. Further studies with larger and more diverse populations are essential to validate these findings and identify effective strategies for enhancing knowledge about monkeypox.
